# DNA Methylation Analyses Unveil a Regulatory Landscape in the Formation of Nacre Color in Pearl Oyster *Pinctada fucata martensii*

**DOI:** 10.3389/fgene.2022.888771

**Published:** 2022-06-13

**Authors:** Ziman Wang, Shaojie Zhu, Shixin Yin, Zihan Zhao, Zhe Zheng, Yuewen Deng

**Affiliations:** ^1^ Fisheries College, Guangdong Ocean University, Zhanjiang, China; ^2^ Guangdong Science and Innovation Center for Pearl Culture, Zhanjiang, China; ^3^ Guangdong Provincial Engineering Laboratory for Mariculture Organism Breeding, Zhanjiang, China; ^4^ Guangdong Provincial Key Laboratory of Pathogenic Biology and Epidemiology for Aquatic Economic Animals, Zhanjiang, China

**Keywords:** epigenetic regulation, DNA methylation, nacre color, carotenoids, *Pinctada fucata martensii*

## Abstract

Pearl color is regulated by genetics, biological pigments, and organic matrices and an important factor that influences the pearl economic value. The epigenetic regulation mechanism underlying pearl pigmentation remains poorly understood. In this study, we collected the mantle pallial (MP) and mantle central (MC) of the golden-lipped strain, and MP of the silver-lipped strain of pearl oyster *Pinctada fucata martensii*. The whole-genome bisulfite sequencing (WGBS) technology was employed to investigate the possible implication of epigenetic factors regulating nacre color variation. Our results revealed approximately 2.5% of the cytosines in the genome of the *P*. *fucata martensii* were methylated, with the CG methylation type was in most abundance. Overall, we identified 12, 621 differentially methylated regions (DMRs) corresponding to 3,471 DMR-associated genes (DMGs) between the two comparison groups. These DMGs were principally enriched into KEGG metabolic pathways including ABC transporters, Terpenoid backbone biosynthesis, and fatty acid degradation. In addition, integrating information about DMGs, DEGs, and function annotation indicated eight genes LDLR, NinaB, RDH, CYP, FADS, fn3, PU-1, KRMP as the candidate genes related to pigmentation of nacre color. A further study proved that the pigment in nacre is violaxanthin. The results of our study provide the support that there is an association between nacre color formation and DNA methylation profiles and will help to reveal the epigenetic regulation of nacre pigmentation formation in pearl oyster *P*. *fucata martensii*.

## Introduction

The diverse forms and alternative colors of mollusk shells have been the focus of scientists for hundreds of years. The color variation of bivalve mollusks shells has also been extensively studied in economical species, such as scallops (Ding et al., 2015; Zhu et al., 2021), mussels (Li et al., 2014), clam (Nie et al., 2020; Ding, 2021), and oyster (Han et al., 2020; He et al., 2022). Several studies have been conducted to reveal the mechanisms of shell color formation and have identified multiple factors, involved in environmental factors and genetic factors, which contribute to the variability of mollusk shell color. In general, shell color is associated with the presence of shell pigments, mainly melanin, carotenoids, porphyrins, and bile pigments (Wang et al., 2020). The striking color range of some gem-producing Mollusca, such as pearl oysters, has also attracted attention (Williams, 2017), color in pearl oysters is of importance for pearl production. It has been proposed that nacre color formation is under to complex control of multiple factors, not only through the biological pigments (melanin and carotenoids), organic matrices, and metal ions but also through genetic regulation (Wang et al., 2020). However, the underlying regulatory mechanisms remain unclear.

Epigenetics refers to processes of heritable changes in gene activity without manipulating the underlying DNA sequence (Turner, 2009), including DNA methylation, histone modifications, and non-coding RNA activity (Gavery and Roberts, 2010). Epigenetic modifications can have an impact on gene transcription and expression. DNA methylation was the first epigenetic modification to be identified and has been extensively studied in eukaryotes. They are involved in many biological processes, such as maintaining the normal cell function of higher organisms, genetic markers, growth and development, and other biological functions (Akalin et al., 2012; Gavery and Roberts, 2013), and play an essential role in the process of pigmentation (Zhang et al., 2017). It has been shown that methylation modification is one of the causes of body color variations in aquatic animals. For example, Li et al. (2015) showed that DNA methylation levels of DEGs were negatively correlated with gene expression in red skin and white skin of koi carp, suggesting that DNA methylation regulates gene expression and thus affects coloration. Lv et al. (2013) conducted the DNA methylation analyses of adductor muscle for “Haida golden scallop” and common ones of scallop, and the results showed that the levels of DNA methylation levels of two selected different methylation sites correlated with regulating carotenoid accumulation. Yuan et al. (2021) identified differently methylated regions related to shell color between brown and white shells, which were primarily enriched in GO functions and pathways relevant to melanin and porphyrin biosynthesis (Yuan et al., 2021). In pearl oyster *Pinctada margaritifera*, Stenger et al. (2021) explored how methylation modifies the color change of the nacre under the change of depth at which an oyster is grown. It is found that the methylation patterns of the four different patterns of clams are different, which may be related to the shell color patterns. A scrutiny of the above research suggest that methylation modifications are crucial in genetic studies of body coloration in an animal.

The pearl oyster *Pinctada fucata martensii* is an economically important species of marine pearl culture. The biomineralization process of shell in pearl oysters is directed by the epithelial cells of the mantle tissue, which differs in structure and function in different parts of the mantle tissue (Zhang et al., 2020). The pallial and central mantle contributes to the secretion of the nacre. pallial mantle, with ciliated epithelium and epithelial secretory cells, may carry out absorbing of deposited particles (Parvizi et al., 2017). We have developed golden- and silver-lipped lines of pearl oyster *P*. *fucata martensii* after three successive selections for nacre color in a base stock. Interestingly, the area covered by the pallial mantle area is golden, while the area covered by the central mantle is silver in golden-lipped pearl oysters, and the whole inner shell is silver in silver-lipped pearl oysters. In the present study, whole-genome bisulfite sequencing (WGBS) was conducted to identify the DNA methylation patterns in mantle tissues of golden- and sliver-lipped pearl oysters, leading to the identification of candidate genes responsible for nacre coloration. This work provides the basis for speculation on the epigenetic mechanisms responsible for color formation in the nacre color and provides a reference for developing breeding programs.

## Materials and Methods

### Experimental Animals and Sample Collection

**FIGURE 1:**
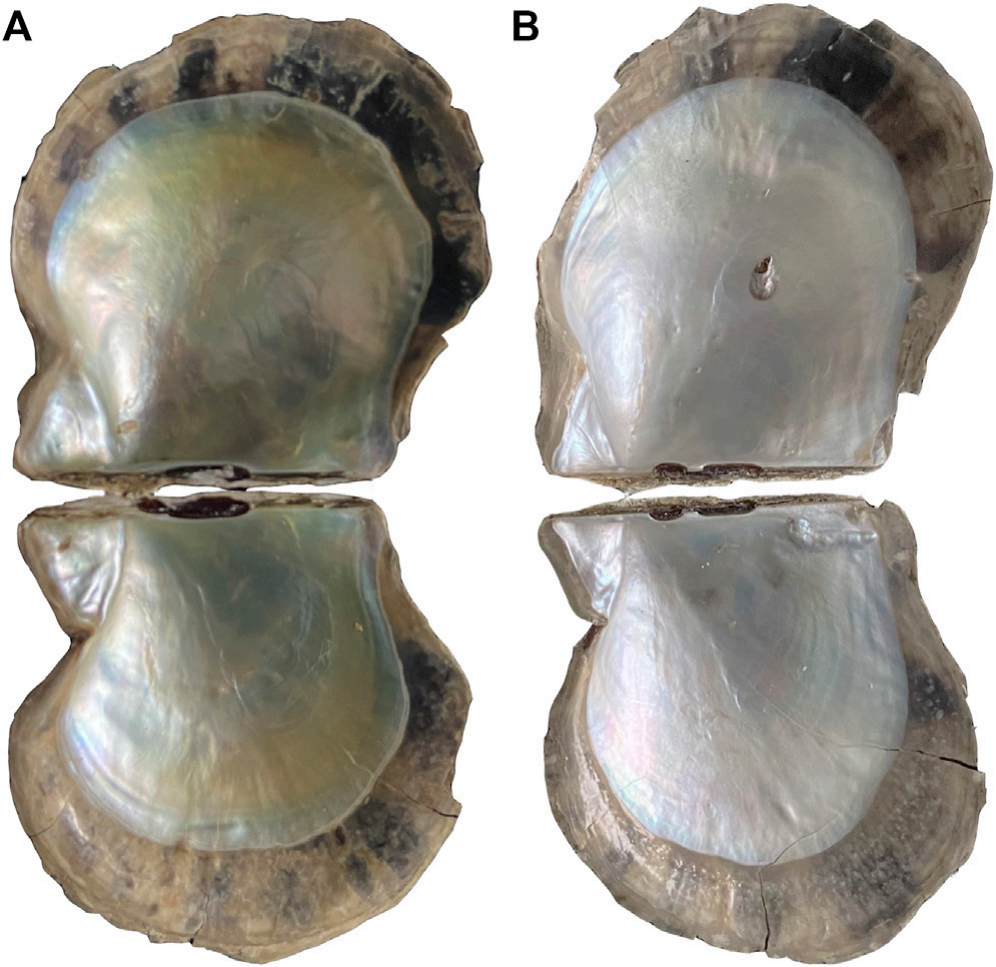
**(A)** Golden-lipped pearl oyster; **(B)** sliver-lipped pearl oyster.

The golden- and silver-lipped lines were farmed in the sea area of Leizhou (Zhanjiang City, Guangdong Province, China). The color of the nacre at the flat position of the left shell was measured (Figure 1) using a multifunctional colorimeter (NR60CP, 3NH, Shenzhen, China). The visible reflection spectrum was obtained through a LED light as an illumination source (illuminant D65, 8° view angle, illumination area diameter 4 mm), and a white porcelain standard plate was used for calibration. The color parameters were calculated and returned from the spectra. The color parameters are calculated and returned from the spectrum. The device calculates and returns the color coordinates of the parameters CIELAB L*, a*, b*, from the spectrum and calculates the color difference value ΔE. Mantle pallial (MP) and mantle central (MC) were sampled and then frozen in liquid nitrogen.

### Raman Spectroscopy to Detect Pigments in Shell Nacre

Raman scattering spectra were obtained on a LabRAM HR Raman microspectrometer (Horiba Scientific, Tokyo, Japan) with 514 nm solid-state laser (Spectra-Physics) excitation, an appropriate wavelength for RRMS for polyenes, including carotenoids. The grating line density was 1800 gr/mm, the exposure time was 3 s, and the acquisition resolution was 0.7 cm^−1^. Raman spectra from three regions of shell surface for each specimen to verify that the structures of the pigments did not vary locally within the same color region.

### Quantitative and Qualitative Evaluation of Carotenoids by HPLC

The carotenoids of nacre were characterized and quantified using HPLC. The nacreous layers were obtained by removing periostraca and prismatic layers off the shells using a polisher. Then nacres were machine-powdered to a particulate size of ∼200 μm individually, and 0.5 g of each sample was taken.0.1% butylated hydroxytoluene (BHT) was added to 2 ml of absolute ethanol and water bath at 80°C for 5 min, then, 100 μL of potassium hydroxide solution (80% w/v) was added to water bath for 15 min. Following, 1 ml of purified water and 1 ml of n-hexane were added and centrifuged at 3000 rpm for 5 min, the upper phase was collected. Twice of the extracts were combined and dried using nitrogen. Next, the crude extract was dissolved in 0.2 ml of methanol solution. The filtered samples were automatically loaded into the Thermo DGLC Dual Ternary UHPLC System, which contained a YMC C30 Carotenoid column (150*4.6 mm, 3 μm), at a flow rate of 1 ml/min. Quantification of carotenoids was performed using external standards. Data analysis was performed with Chromeleon7 software.

### DNA and RNA Isolation

Nine samples were used for DNA and RNA isolation, including three MP tissues (YMP) and three MC tissues (YMC) of golden-lipped pearl oysters, and three MP tissues (WMP) of sliver-lipped pearl oysters, respectively. Genomic DNA was isolated from mantle tissue using TIANamp Marine Animals DNA Kit (TIANGEN BIOTECH, Beijing, China), according to the manufacturer’s protocols. DNA concentration and integrity were checked by SimpliNan and 1% agarose gel electrophoresis, respectively. Following the manufacturer’s protocol, total RNA was isolated using a Trizol reagent kit (Thermo Fisher Scientific, MA, United States). RNA quantification and integrity were assessed on an Agilent 2100 Bioanalyzer (Agilent Technologies, Palo Alto, CA, United States).

### BS Library Construction, Sequencing, and Mapping

Genomic DNAs were fragmented by Sonication (Covaris, Massachusetts, United States) to a length of 100–300 bp. After purification using MiniElute PCR Purification Kit (QIAGEN, MD, United States), the fragmented DNAs were end-repaired, and then a single “A” nucleotide was added to the 3’ end. Subsequently, the genomic fragments were ligated to methylated sequencing adapters and treated with bisulfite converted using a Methylation-Gold kit (ZYMO, CA, United States). Finally, PCR amplification was performed, and sequencing was performed using Illumina HiSeqTM 2500 by Gene Denovo Biotechnology Co. (Guangzhou, China).

The raw reads were obtained after quality filtering and adaptor trimming. Reads containing more than 40% low-quality bases (Q-value ≤ 20) and more than 10% unknown nucleotides (N) were removed. The obtained clean reads were mapped to the *Pinctada facata martensii* genome using BSMAP software (Xi and Li, 2009) (version: 2.90) by default. Methylated cytosines were then called using a custom Perl script and tested for methylated cytosines using the correction algorithm described in Lister R.et al. (2009).

### Methylation Analysis

We analyzed the methylation level based on methylated cytosine percentage in the whole genome and in different regions of the genome for each sequence context (CG, CHG, and CHH). The average methylation levels of each type of C base (CG, CHG, and CHH) were counted for different regions of the encoded genes (containing gene body, exon, intron, CDS, 5′ UTR and 3′ UTR, upstream_2k, downstream_2k), and the methylation levels of the flanking 2 kb regions and gene bodies were plotted based on the average methylation levels of each window.

### Identification of Differentially Methylated Regions and Function Analysis of Differentially Methylated Region-Associated Genes

We used a 200 bp window in a genome-wide scan to calculate the mean DNA methylation rate within each window and compare the differences in methylation levels between samples within each window to identify differentially methylated regions (DMRs) between two samples. For all C, CG, CHG, and CHH, we performed differential methylation analysis separately and filtered using different criteria: 1) For CG, numbers of GC in each window ≥5, the absolute value of the difference in methylation ratio ≥0.25, and q ≤ 0.05; 2) For CHG, numbers in a window ≥5, the absolute value of the difference in methylation ratio ≥0.25, and q ≤ 0.05; 3) For CHH, numbers in a window ≥15, the absolute value of the difference in methylation ratio ≥0.15, and q ≤ 0.05; 4) For all C, numbers in a window ≥20, the absolute value of the difference in methylation ratio ≥0.2, and q ≤ 0.05.

DMR-associated genes (DMGs) were analyzed based on DMRs overlapping with functional regions of genes, followed by gene ontology (GO) and Kyoto Encyclopedia of Genes and Genomes (KEGG) enrichment analysis of DMGs using GOseq R software and KOBAS software. (Kanehisa and Goto., 2000).

### RNA-Seq Library Generation, Sequencing, and Mapping

The mRNA was enriched with Oligo (dT)beads and fragmented into short fragments using fragmentation buffer, then reverse transcribed into cDNA using random primers. Second-strand cDNA was synthesized using DNA polymerase I, RNase H, dNTP, and buffer and purified using QiaQuick PCR extraction kit (Qiagen, Venlo, Netherlands), and the purified second-strand cDNA was end-repaired by adding A bases and ligated to Illumina sequencing adapters. Finally, the ligation products were size selected by agarose gel electrophoresis, PCR was amplified, and sequencing was carried out for different libraries using Illumina Novaseq6000 by Gene Denovo Biotechnology Co (Guangzhou, China).

### Differential Expression Genes and Enrichment Analysis

After raw data filtering and mapping to *Pinctada fucata martensii* genome, FPKM values were used to quantify gene expression abundance and variation. Differential expression analysis of three groups was performed using the DEGseq R package (1.20.0). The thresholds for DEGs were set as corrected *p*-value < 0.05 and |log 2 (fold change)|> 1using Benjamini and Hochberg method (Benjaminiand and Hochberg, 2000). GO enrichment analysis and KEGG pathways analysis of DEGs were implemented.

### Correlation of Differentially Methylated Regions and Differential Expression Genes

In order to investigate the effect of DNA methylation on gene expression in different mantle tissues, we categorized the genes into four classes, including a non-methylation group, a low methylation group, a middle-methylation group, and a high methylation group. The relationships between DNA methylation and gene expression within the ±2 kb flanking regions and gene body region were discerned statistically using Spearman correlation analysis.

### Quantitative Real-Time PCR Validation

Quantitative real-time PCR (qRT-PCR) was conducted using the SYBR^®^ qPCR Master Mix (TransGen Biotech, Beijing, China) on the LightCycler 480 real-time PCR instrument (Roche Diagnostics, Burgess Hill, United Kingdom). Amplification parameters refer to Wang et al., 2019. The specific primers were listed in Supplementary Table S3.

## Results

### Phenotypes of Nacre Color

In order to measure the nacre color, we measured the lightness (L), red/green (a), and yellow/blue (b) strengths from nacre using the International Commission on illumination (CIE) model. We focus on the value of b, where higher values indicate greater yellow coloration. The b value of the region in MP of golden-lipped pearl oysters is significantly higher than silver-lipped pearl oysters (*p* < 0.05). In addition, the b value of the region in MP is significantly higher than MC in the golden-lipped pearl oysters (*p* < 0.05), and it has no significant difference between the two regions in the silver-lipped pearl oysters (*p* > 0.05) (Table 1).

**TABLE 1:** The color parameters of the nacre area covered by MP and MC tissues.

Sample	L^∗^	a^∗^	b^∗^	ΔE^∗^
YMP	79.66 ± 4.05^a^	−4.21 ± 1.15^a^	9.23 ± 0.78^a^	17.82 ± 3.66^a^
YMC	81.37 ± 3.30^a^	−0.95 ± 2.26^b^	2.18 ± 0.58^b^	13.47 ± 3.41^b^
WMP	83.33 ± 4.53^a^	−3.46 ± 1.74^ab^	−0.25 ± 2.67^c^	12.34 ± 4.44^b^
WMC	81.89 ± 3.730^a^	−1.63 ± 1.52^b^	0.04 ± 0.69^bc^	13.03 ± 3.44^b^

The superscripts of different letters in the table indicate significant differences, *p* < 0.05.

### The Pigments of Nacre Color

**FIGURE 2:**
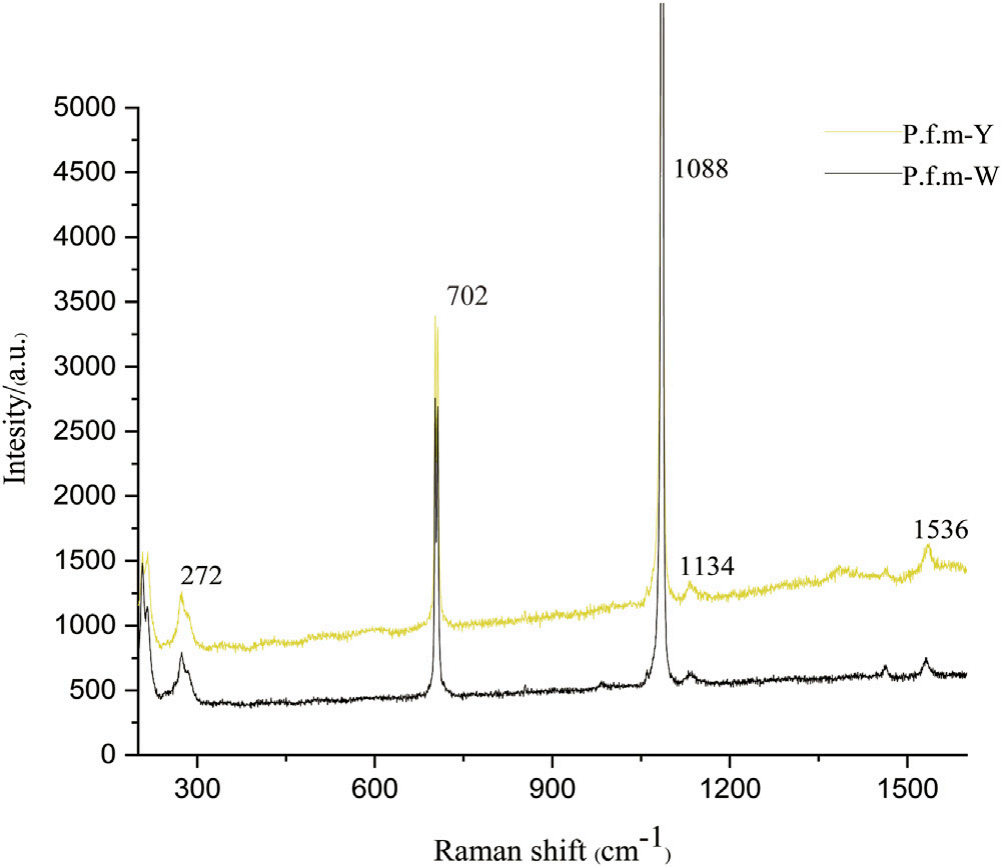
Raman spectra of nacre of *Pinctada fucata martensii*.

A spike peak at 1088 cm^−1^ was allocated to the symmetric CO_3_^2−^ stretching mode of CaCO_3_in the shell. 272 cm^−1^ and 702 cm^−1^ were also identified as aragonite crystal. The same peaks were observed in all measured samples. Two major bands (ca. 1134 and 1536 cm^−1^) were observed from the pigmented area of the same specimens (Figure 2). The two main bands at 1100–1150 and 1500–1550 cm^−1^represent the C─C (ν2) and C═C (ν1) stretching vibrational modes, respectively. Those observations revealed that the pigment has a very similar chemical structure to carotenoids.

### Quantitative and Qualitative of Carotenoids in Nacre

**FIGURE 3:**
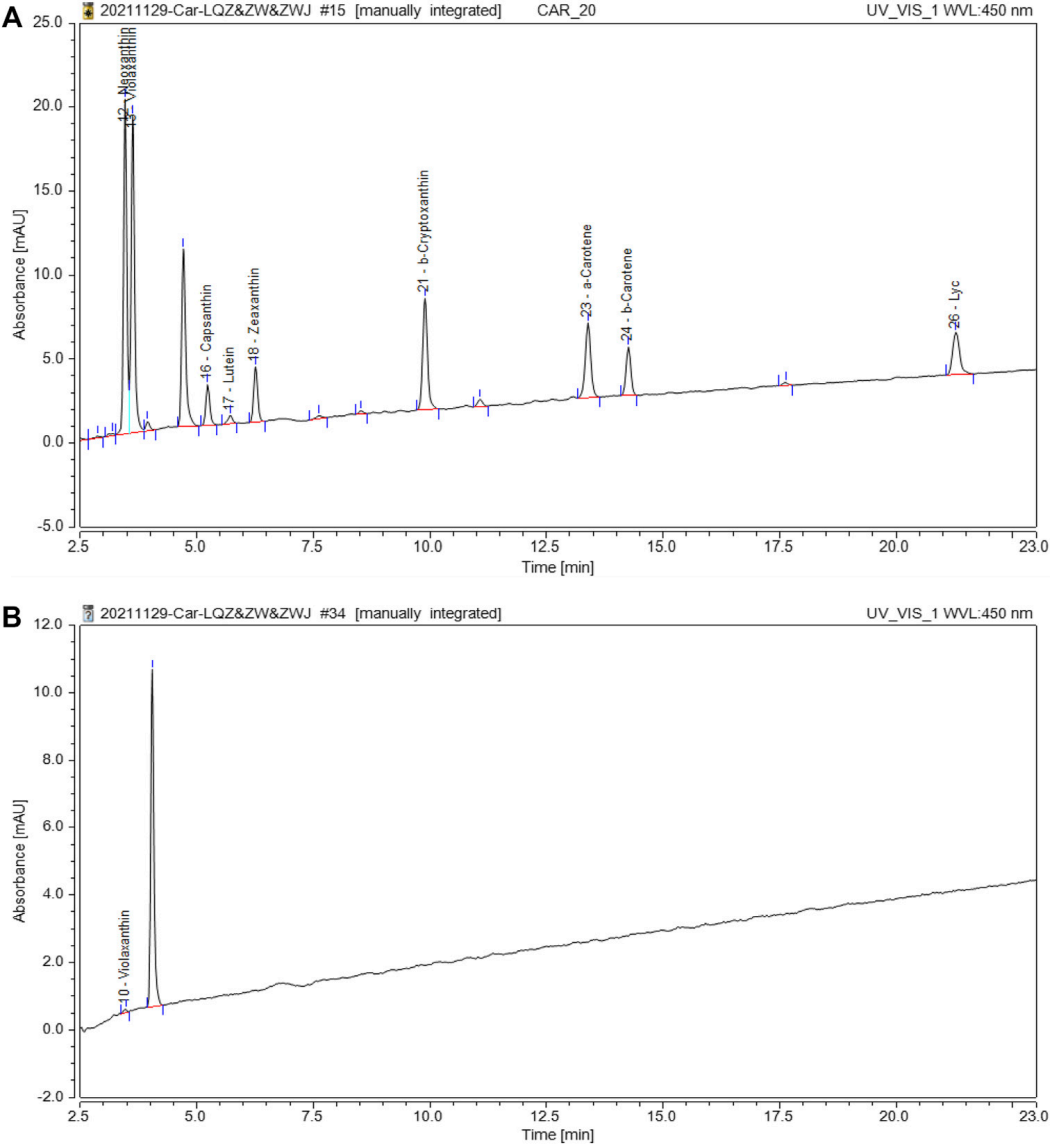
HPLC separation of carotenoids in nacre. **(A)**: The chromatogram of the carotenoid standard; **(B)**: The chromatogram of carotenoids in shell nacre sample.

To determine whether carotenoids were related to the nacre color, the concentration of carotenoids was checked by HPLC in the nacre. According to the retention time of the peak in comparison with the standard product, the carotenoid in nacre was found to be violaxanthin (Figure 3), and the relative content was 0.0009 µg/100 mg in golden nacre.

### DNA Methylation Mapping, Patterns, and DNA Methylation Levels Analysis

In order to explore the effect of DNA methylation profile in the formation of nacre color, the single-base resolution maps of DNA methylation were generated of mantle tissues of YMP, YMC, and WMP. The Bisulfite conversion efficiency (BCE) of all samples exceeded 98.90%. After data filtering, over 295 million high-quality clean reads were gained in each group, the Q20 value was more than 96%, and detected in all chromosomal regions. The sequencing depth per sample compared to the reference genome is more than 20× per strand for each sample. The reference genome mapping rate ranged from 63.51 to 65%, indicating that the data could be available for subsequent analysis (Table 2). An average methylation level of the whole genome is 2.39% of genomic cytosines, 12.76, 0.77, and 0.78% were CG, CHG, and CHH types, respectively. To investigate the function of methylation in transcriptional regulation, we examined the DNA methylation levels in different functional elements of the genome (including genebody, exon, intron, CDS, 5′UTR, 3′UTR, upstream 2k, downstream 2k) in different sequence contexts. For all methylation types (C, CHG, and CHH), similar methylation levels were observed in each functional region for the 5mC in nine samples. The DNA methylation levels were highest in 3′ UTR, followed by exon, CDS, intron, and 5′ UTR, and lowest in the promoter region (Supplementary Table S1). Overall, these samples had similar CpG methylation profiles at the genome-wide level.

**TABLE 2:** Read quality and genome coverage of WGBS.

Sample	BCE(%)	Clean reads	Mapping rate (%)	Sequence depth	C (%)	CG (%)	CHG(%)	CHH(%)
YMP1	99.03	257407838	64.95	25.39	2.33	12.45	0.76	0.76
YMP2	99.02	257124096	65.00	25.38	2.33	12.51	0.75	0.75
YMP3	99.01	271793294	64.66	26.69	2.44	13.26	0.76	0.76
YMC1	99.01	262048260	63.51	25.28	2.37	12.65	0.77	0.78
YMC2	99.00	271775282	64.81	26.75	2.36	12.62	0.76	0.76
YMC3	99.01	240688780	63.26	23.13	2.34	12.29	0.77	0.79
WMP1	99.00	294883988	64.57	28.92	2.39	12.77	0.77	0.78
WMP2	98.90	243861722	64.41	23.85	2.49	13.24	0.83	0.83
WMP3	98.97	211141580	64.63	20.72	2.43	13.02	0.79	0.79

### Identification of Differentially Methylated Regions

Differentially methylated regions (DMRs) were used to quantitatively characterize the differences in DNA methylation between samples. To characterize the change of DNA methylation in mantle tissues, we use a 200 bp window to scan the whole genome to identify DMR and compare the DNA methylomes of WMP vs. YMP, and YMC vs. YMP. A total of 10,015 DMRs were identified in WMP vs. YMP comparison, in CG type, compared with the YMP group, the WMP group showed 4,722 up-regulated DMRs and 5,252 down-regulated DMRs. In order to further characterize the DMRs, we analyzed and annotated DMR-related genes. The DMRs were aligned to different genomic elements, mainly in the gene body, the DMRs corresponding to 2,860 genes. Totally, 2,606 DMRs were identified in YMC vs. YMP comparison group, in CG type, compared with the YMP group, the YMC group showed 1,395 up-regulated MDRs and 1,131 down-regulated DMRs, and the DMRs corresponding to 1007 genes (Supplementary Figure S1).

### Functional Enrichment Analysis of Differentially Methylated Region-Associated Genes

**FIGURE 4:**
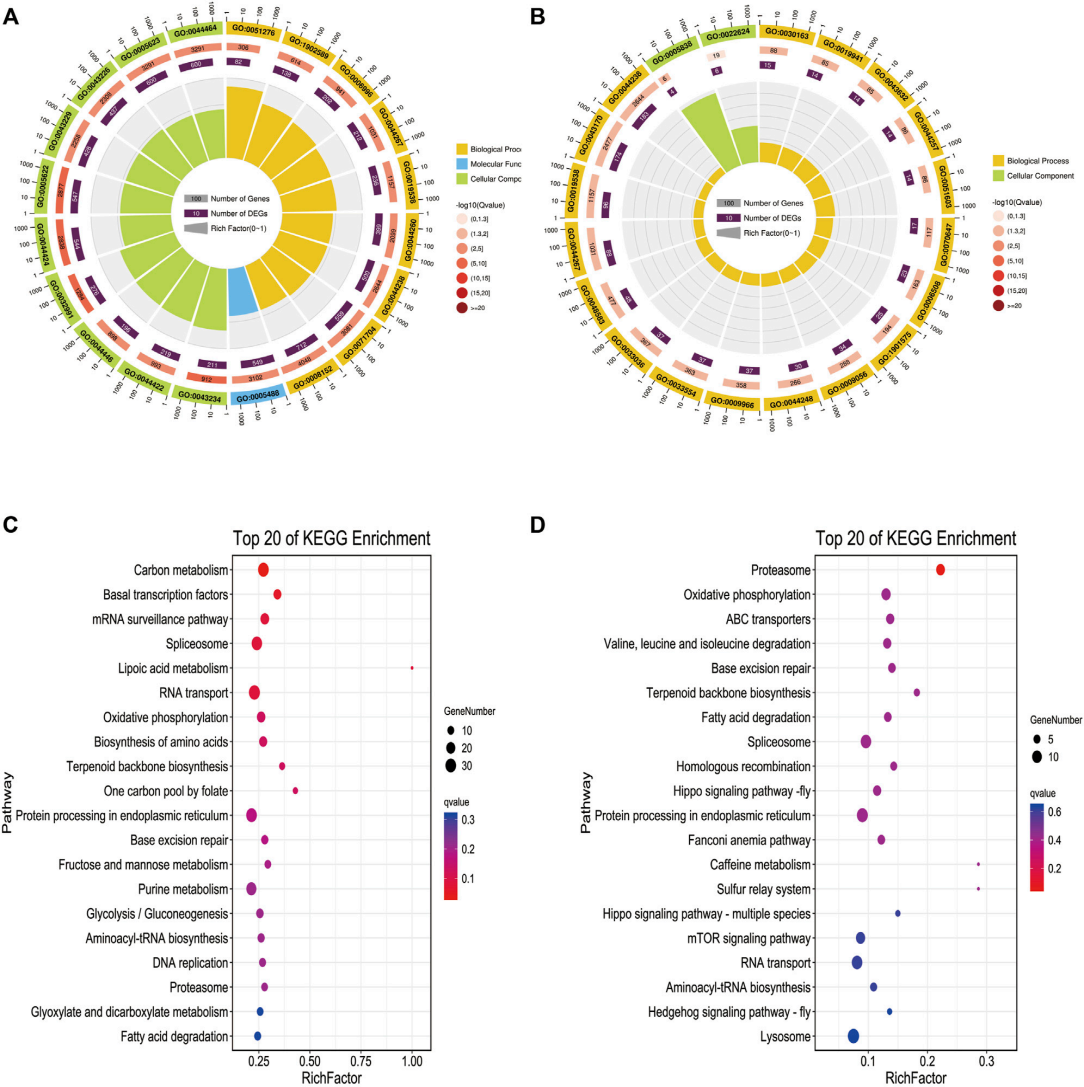
GO and KEGG function enrich in DMR-related genes. **(A)** Statistics of the top 20 GO terms enriched for DMGs in YMP vs. WMP; **(B)** statistics of the top 20 GO terms enriched for DMGs in YMP vs. YMC; **(C)** statistics of the top 20 pathways enriched for DMGs in YMP vs. WMP; **(D)** statistics of the top 20 pathways enriched for DMGs in YMP vs. YMC.

To further explore the biological function of these methylated regions, we conducted GO and KEGG pathway analyses to annotate the DMGs. In WMP vs. YMP group, the GO analysis showed that the DMGs were enriched in biological processes including primary metabolic process, chromosome, organization, organelle organization, cellular macromolecule, metabolic process, and single-organism organelle organization, etc. (Figure 4A). In the YMP vs. YMC group, DMGs were involved in biological processes including catabolic process, cellular protein metabolic process, proteolysis, protein metabolic process, etc. (Figure 4B). In the WMP vs. YMP group, the KEGG analysis suggested that the DMGs were involved in the carbon metabolism, basal transcription factors, mRNA surveillance pathway, spliceosome, lipoic acid metabolism, RNA transport, etc. (Figure 4). In the YMP vs. YMC group, the DMGs were enriched in the proteasome, oxidative, phosphorylation, ABC transporters, valine, leucine, and isoleucine degradation, base excision repair, terpenoid backbone biosynthesis, and fatty acid degradation, etc. (Figure 4D).

### Data Analysis of RNA-Seq

Based on Illumina sequencing and after filtering low quality and short sequences, a total of 42.88M raw sequence reads were obtained from YMP, YMC, and WMP groups. Over 97% of clean reads were obtained after filtering. Of these clean reads, more than 68% of reads were uniquely mapped to the *Pinctada fucata martensii* reference genome, in a total of 21,542 known mRNAs and 3,777 novel mRNAs were identified. These data are listed in Table 3.

**TABLE 3:** Information and quality of RNA-seq.

Sample	Raw reads	Clean reads	Q20 (%)	Q30 (%)	GC (%)	Unique_Mapped (%)
WMP-1	41239682	40497034	97.45%	92.93%	46.28%	68.94%
WMP-2	40622248	39864594	97.52%	93.11%	45.31%	73.54%
WMP-3	38714112	38129994	97.38%	92.75%	44.26%	73.68%
YMP-1	37829736	36916170	97.48%	93.00%	45.66%	69.45%
YMP-2	38343990	37790198	97.60%	93.29%	45.63%	71.78%
YMP-3	39151150	38487494	97.42%	92.84%	45.83%	73.82%
YMC-1	38695014	38104662	97.31%	92.56%	41.92%	71.44%
YMC-2	40294512	39771026	97.43%	92.85%	42.27%	72.39%
YMC-3	42678394	42096998	97.29%	92.50%	41.70%	73.02%

### Identification of Differentially Expressed Genes and Enrichment Analyses

**FIGURE 5:**
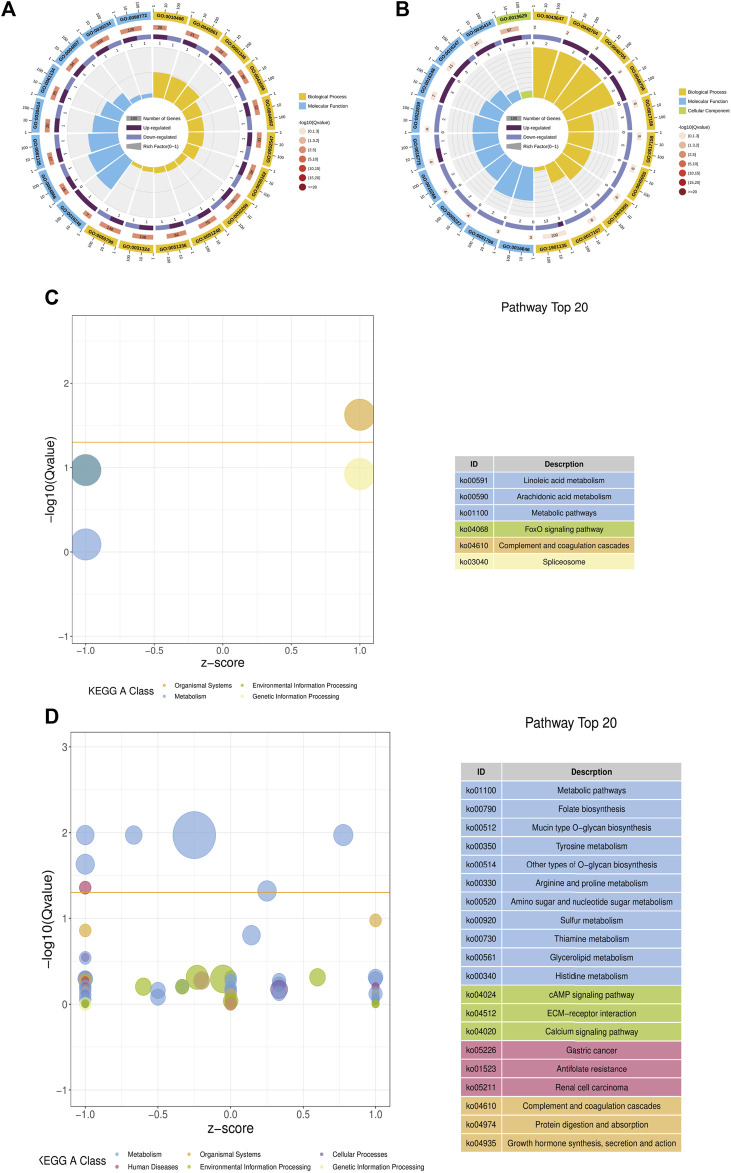
GO and KEGG function enrich in DEGs. **(A)** Statistics of the top 20 GO terms enriched for DEGs in YMP vs. WMP; **(B)** statistics of the top 20 GO terms enriched for DEGs in YMP vs. YMC; **(C)** statistics of the pathways enriched for DEGs in YMP vs. WMP; **(D)** statistics of the top 20 pathways enriched for DEGs in YMP vs. YMC.

In YMP vs. WMP group, compared with YMP, 21 DEGs were identified, of which 12 were up-regulated and 9 were down-regulated. In YMP vs. YMC group, compared with YMP, 895 DEGs were obtained, including 367 up-regulated genes and 528 downregulated genes (Supplementary Figure S2). Gene Ontology (GO) and Kyoto Encyclopedia of Genes and Genomes (KEGG) analyses were conducted to elucidate the potential functions of DEGs. In YMP vs. WMP, DEGs related to biological processes and molecular function were significantly enriched (Figure 5A). The category of biological processes, which include negative regulation of peptidase activity, proteolysis, hydrolase activity, catalytic activity, and cellular protein metabolic process, was significantly enriched. The category of molecular function includes endopeptidase inhibitor activity, endopeptidase regulator activity, peptidase inhibitor activity, peptidase regulator activity, and enzyme inhibitor activity. In YMP vs. YMC, the GO terms of the actin cytoskeleton, channel regulator activity, channel inhibitor activity, calcium ion regulated exocytosis, regulation of calcium ion-dependent exocytosis, and skeletal system morphogenesis were enriched (Figure 5B). The KEGG analysis indicated that 21 DEGs in YMP vs. WMP group were enriched in 6 pathways, including complement and coagulation cascades, linoleic acid metabolism, FoxO signaling pathway, arachidonic acid metabolism, spliceosome, and metabolic pathways (Figure 5C). In YMP vs. YMC comparison, the pathways of metabolic pathways, mucin type O-glycan biosynthesis, folate biosynthesis, tyrosine metabolism, other types of O-glycan biosynthesis, gastric cancer, arginine, and proline metabolism were enriched (Figure 5D).

### Correlation of Differentially Methylated Regions and Expression of mRNA

**FIGURE 6:**
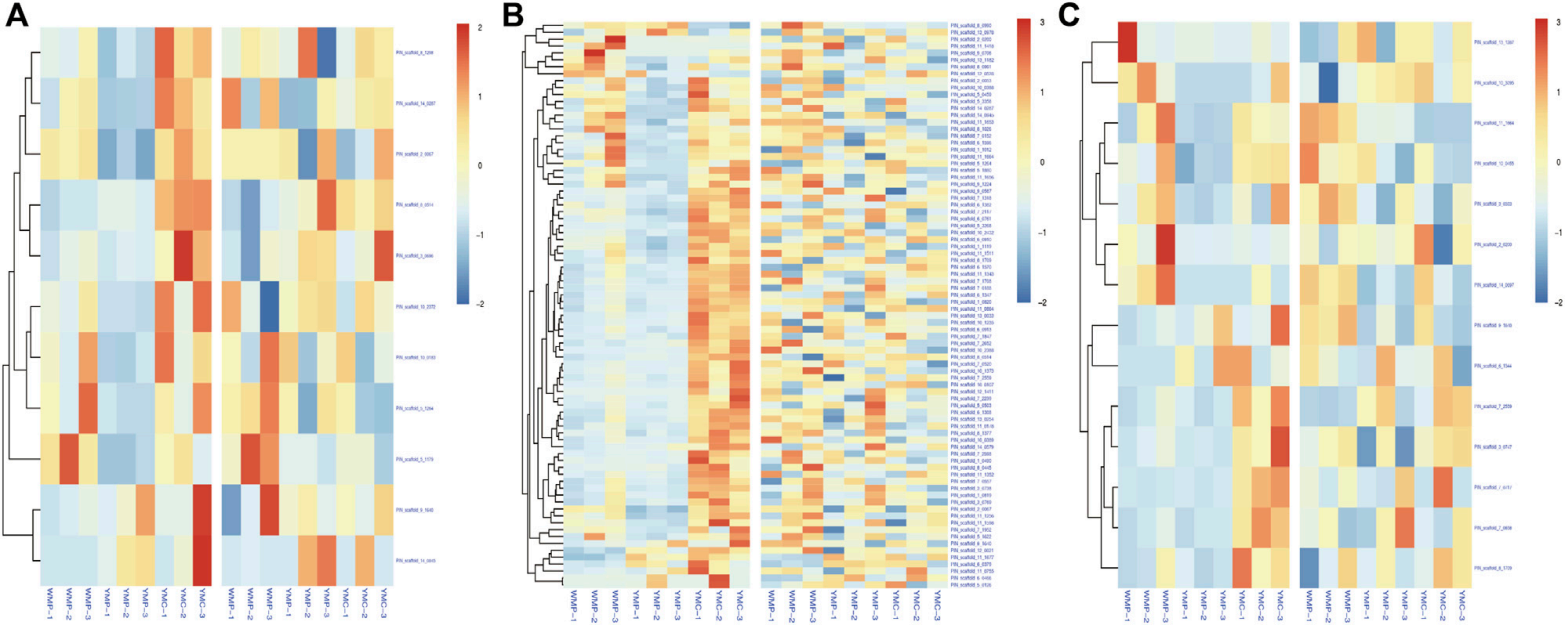
Correlation of DMR-related genes and DEGs. **(A)** Up-stream 2k; **(B)** genebody; **(C)** down-stream 2k.

To investigate the effect of DNA methylation variations on gene expression, we focused on the analysis of the common genes between DMGs and DEGs. Among them, 97 functional genes were differentially methylated and differentially expressed in the two comparative groups simultaneously (Figure 6). In the WMP vs. YMP group, 27 up-regulated DMRs and gene expressions were positively correlated, 50 were negatively correlated with gene expression, 19 down-regulated DMR and gene expression were negatively correlated, and 66 were positively correlated with gene expression. In the YMC vs. YMP group, 1 up-regulated DMR and gene expression were positively correlated, 4 were negatively correlated with gene expression, 46 down-regulated DMR and gene expression were negatively correlated, and 32 were positively correlated with gene expression. These data suggested that relatively more genes had a positive correlation between expression and methylation. The genes potentially involved in the coloration of nacre were identified in DMRs and are listed in Supplementary Table S2. Carotenoid isomerooxygenase-like is the primary regulator of carotenoid metabolism. There was a DMR detected in the gene of carotenoid isomerooxygenase-like, and compared to the YMP group, methylation is up-regulated in the WMP group. The genes retinol dehydrogenase 12 isoform X1, low-density lipoprotein receptor-related protein 2-like, fatty acid desaturase 2-like isoform X2, and cytochrome P450 2J2-like are involved in carotenoid synthesis and metabolism. They were all differentially methylated between the WMP vs. YMP and YMP vs. YMC comparison groups. Several shell matrix proteins (SMPs) such as fibronectin type III domain-containing protein 2, P-U1, and lysine-rich matrix protein 4, were differentially methylated among YMP vs. YMC comparison group.

### Verification of Genesinvolved in Nacre Coloration by Quantitative Real-Time PCR

Six genes associated with nacre coloration were randomly selected to verify the reliability of the RNA-seq data. The results showed that the expression characterization of these genes was similar to that detected with RNA-seq (Supplementary Figure S3).

## Discussion

DNA methylation plays a critical role in numerous life processes including epigenetic regulatory mechanisms involved in pigmentation (Zhang et al., 2017). It is well known that the genotypes of the same individual are the same, and in the nacre of pearl oysters, the corresponding regions of different mantle regions have different colors. We believe that the methylation difference of mantle may be the reason for the color difference of nacre. It has carried few studies investigating the role of DNA methylation in the nacre coloration of mollusks out. In this study, the DNA methylation patterns and levels in the whole-genome of the *P. f. martensii* were analyzed between silver and gold nacre color individuals, and comparison in different groups was performed to identify DMRs associated with the formation of nacre color. We identified 10015 and 2606 DMRs in two comparison groups, 2860 and 1007 genes related to these DMRs were assigned, respectively.

Many studies have demonstrated that the shell color of mollusks is generally attributable to the presence of biological pigments (Wang et al., 2020). Indeed, yellow color components suspected to be carotenoids have been examined in cultured Akoya gold pearls (Williams, 2017). In the present study, two major bands were detected in nacre at 1100–1150 and 1500–1550 cm^−1^, and the strength in golden nacre is significantly higher than in silver nacre. Polyenes contain conjugated linear carbon-carbon single and double bonds that form a polyene chain. Raman spectroscopy allows the determination of the spectral position associated with the vibrational modes of the C–C single and C=C double bonds. According to previous reports, carotenoids produce characteristic bands in the Raman spectrum at 1000–1020 cm^−1^, 1150–1170 cm^−1^, and 1500–1550 cm^−1^ (Winters et al., 2013). Furthermore, carotenoids were found in nacre by HPLC and characterized as violaxanthin. Similarly, in *C. senatoria*, which has a yellow shell, carotenoids may also be responsible for the coloration (Hedegaard et al., 2006).

Generally, the DNA methylation pattern in invertebrates exhibits a ‘mosaic’, with stretches of methylated DNA interrupted by regions of unmethylated DNA (Gavery and Roberts, 2013). In our study, the DNA methylation profiles were similar in mantle tissues of three groups, about 2.39% of cytosines were detected to be methylated in the genome, which is slightly higher than that of about 1.95% in the Pacific oyster (Hwang et al., 2014) and lower than that in the Eastern oyster (∼2.7%) (Venkataraman et al., 2020) and the Yesso scallop (∼3%) (Yuan et al., 2021). In pearl oysters, the proportion of intragenic methylation is similar to other invertebrates that exhibit predominantly intragenic DNA methylation patterns (Suzuki et al., 2007), the methylated fraction tends to be distributed within the gene bodies, while the gene promoter regions exhibit less methylation (Gavery and Roberts, 2010). In our study, among the 12, 621 DMRs found, 6, 590 (52%) DMRs are located within the gene body. The function of DNA methylation within the gene body is complex, and it has been demonstrated that methylation in this region can regulate the transcription of genes. For instance, DNA methylation in the exon region has been demonstrated to regulate the expression of the phytochrome A gene in *Arabidopsis thaliana* (Chawla et al., 2007). This suggests that the DNA methylation patterns are similar and conserved across species.

To further understand the function of DNA methylation in nacre coloration, the function of DMGs was studied by GO and KEGG enrichment analysis, we found GO terms, namely macromolecular complex binding and lipid binding, which were enriched. Currently, several research studies have demonstrated the coloration of tissues and shells by carotenoids in mollusks, including *Crassostrea gigas* (Maoka et al., 2001), *Hyriopsiscumingii* (Li et al., 2014), and *P. fucata. martensii* (Lei et al., 2017; Zhang et al., 2019). Carotenoids are fat-soluble compounds, and lipid binding is important for the absorption of carotenoids. According to the results of previous studies, the total carotenoid content is strongly correlated with the total lipid content in noble scallops (Zheng et al., 2012; Karsoon et al., 2021). In this study, we found carotenoid isomerooxygenase-like, retinol dehydrogenase 12 isoform X1, low-density lipoprotein receptor-related protein 2-like, fatty acid desaturase 2-like isoform X2, cytochrome P450 2J2-like are differentially methylated. Of these, carotenoid isomerooxygenase-like, retinol dehydrogenase 12 isoform X1 is involved in metabolism process of carotenoids (Lee et al., 2014). These DMGs were mostly distributed in gene body regions of the genome. How methylation variations affect the regulation of function in DMGs needs further study.

Shells are multi-layered structures composed of calcium carbonate crystals together with proteinaceous substances and pigments (Williams, 2017). It has been reported that biomineralization is closely related to shell pigmentation, sequential layer-by-layer mineralization is directed by cells at the edge of the mantle tissues, and pigments in the periostracum layer are produced by the mantle (Xu et al., 2019). In *Pinctada margaritifera*, multiple transcriptome analysis supports the involvement of biomineralization genes in the process of shell pigmentation (Auffret et al., 2020). The shell mineralization process involves energy metabolism and organic matrix crystallization (Zhao et al., 2019; Zhao et al., 2020; He et al., 2021). In the present study, three SMPs, including Fibronectin type III domain-containing protein 2 (FN3), P-U1, and lysine-rich matrix protein 4 (KRMP) (Liang et al., 2015; Liu et al., 2015)were enriched in the DMRs and significantly different expression expressed in YMP vs. YMC comparison group, specifically, the DNA methylation status and mRNA expression levels of P-U1 were negatively correlated, while the positive correlation was detected for the FN3 and KRMP. These results probably indicate that the biomineralization process may be influenced by DNA methylation, and regulated the nacre coloration.

## Conclusion

In summary, WGBS analysis was performed in *Pinctada fucata martensii* with different nacre colors, and DMRs and DMGs in different comparisons were identified. Several related DMRs and genes (LDLR, NinaB, RDH, CYP, FADS, fn3, PU-1, KRMP) were exposed to help us to understand the epigenetic regulation of nacre coloration.

## Data Availability

The datasets presented in this study can be found in NCBI Sequence Read Archive (SRA) under BioProject accession PRJNA818427 and PRJNA820126.

## Supplementary Material

Supplementary Figure S1 Numbers of DMRs are shown for the C, CG, CHG, and CHH.

Supplementary Figure S2 Statistics of differentially expression genes.

Supplementary Figure S3 Genes selected for validation and the fold change in the pairwise comparison for both RNA-seq and RT-PCR analysis.
