# Supplementary figures and images for: DNA Methylation Analyses Unveil a Regulatory Landscape in the Formation of Nacre Color in Pearl Oyster Pinctada fucata martensii
Source: Front Genet. 2022 Jun 13;13:888771. doi: 10.3389/fgene.2022.888771 (PMC9234178; doi:10.3389/fgene.2022.888771)

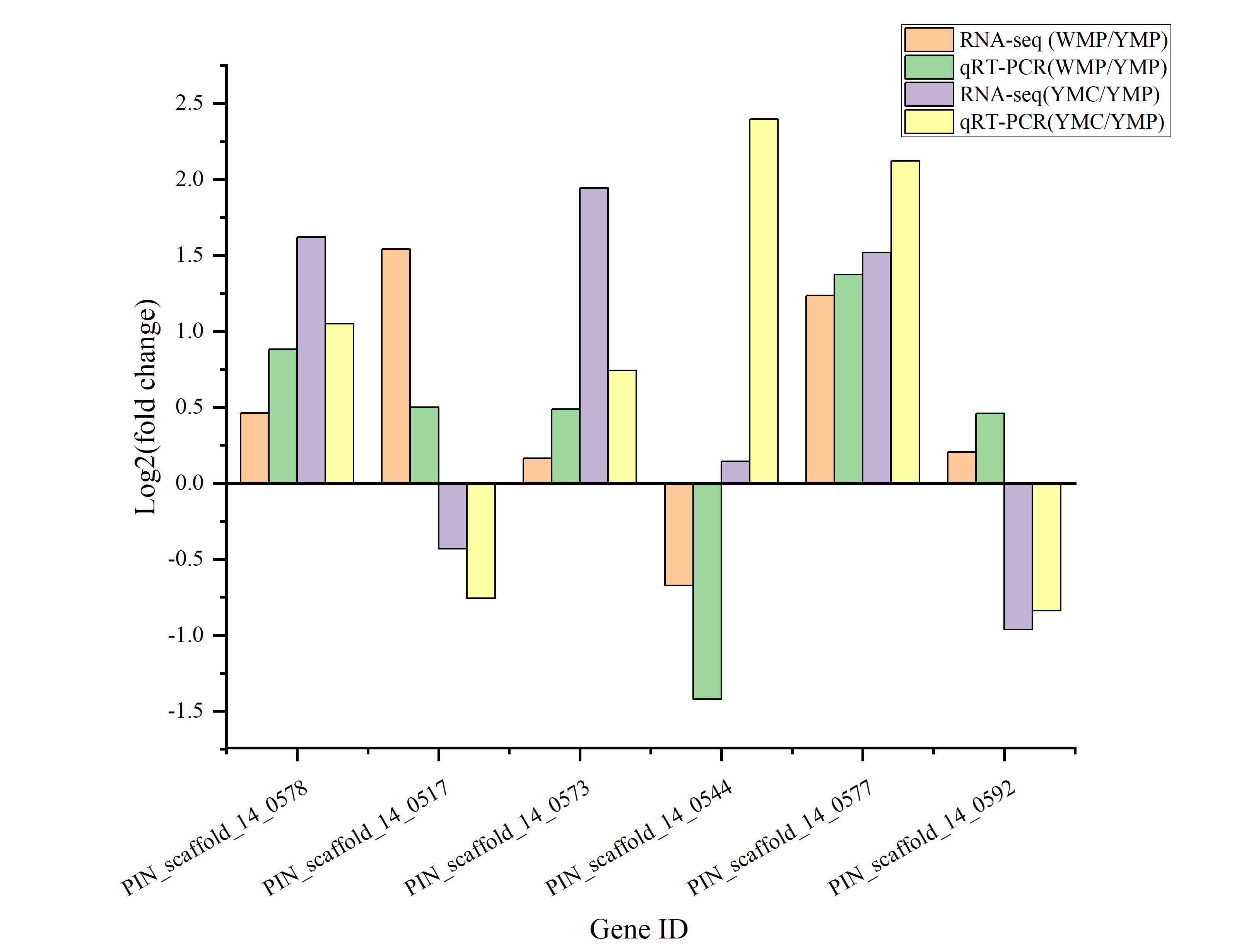

Supplement: Supplementary file 1 [file Image3.jpeg]

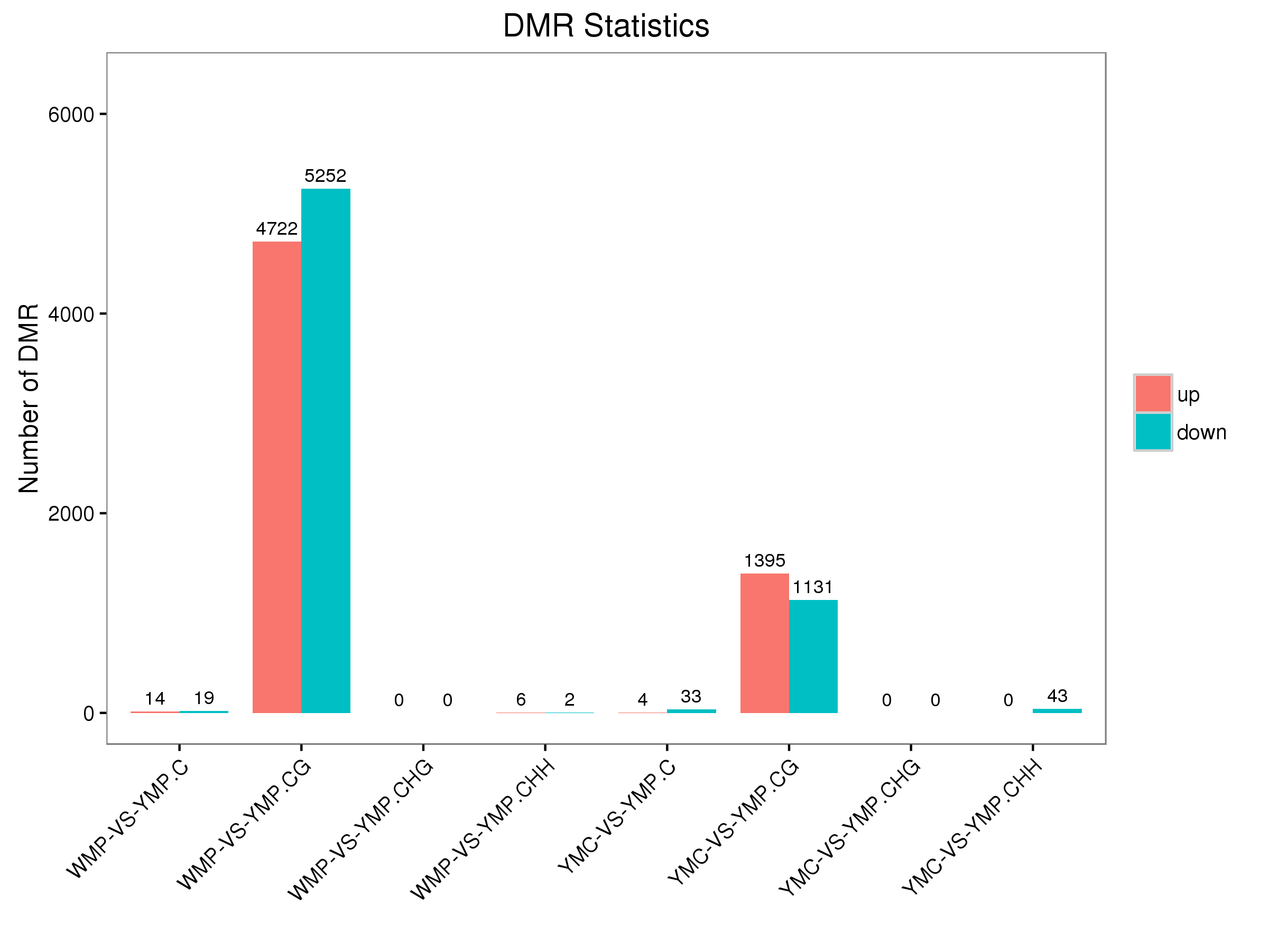

Supplement: Supplementary file 3 [file Image1.TIF]
